# Asynchronous Video Directly Observed Therapy to Monitor Short-Course Latent Tuberculosis Infection Treatment: Results of a Randomized Controlled Trial

**DOI:** 10.1093/ofid/ofae180

**Published:** 2024-03-26

**Authors:** Richard S Garfein, Lin Liu, Javier Cepeda, Susannah Graves, Stacie San Miguel, Antonette Antonio, Jazmine Cuevas-Mota, Valerie Mercer, McKayla Miller, Donald G Catanzaro, Phillip Rios, Fredric Raab, Constance A Benson

**Affiliations:** Herbert Wertheim School of Public Health and Human Longevity Science, University of California, San Diego, California, USA; Division of Infectious Disease and Global Public Health, School of Medicine, University of California, San Diego, California, USA; Herbert Wertheim School of Public Health and Human Longevity Science, University of California, San Diego, California, USA; Department of Epidemiology, Johns Hopkins Bloomberg School of Public Health, Baltimore, Maryland, USA; Tuberculosis Control and Refugee Health Branch, San Diego County Health and Human Services Agency, San Diego, California, USA; Student Health Services, University of California, San Diego, California, USA; Tuberculosis Control and Refugee Health Branch, San Diego County Health and Human Services Agency, San Diego, California, USA; Herbert Wertheim School of Public Health and Human Longevity Science, University of California, San Diego, California, USA; Herbert Wertheim School of Public Health and Human Longevity Science, University of California, San Diego, California, USA; Division of Infectious Disease and Global Public Health, School of Medicine, University of California, San Diego, California, USA; Department of Biological Sciences, University of Arkansas, Fayetteville, Arkansas, USA; Qualcomm Institute, Calit2, San Diego Division, University of California, San Diego, California, USA; Qualcomm Institute, Calit2, San Diego Division, University of California, San Diego, California, USA; Division of Infectious Disease and Global Public Health, School of Medicine, University of California, San Diego, California, USA

**Keywords:** adherence, clinical trial, latent tuberculosis infection (LTBI), mHealth, video directly observed therapy (VDOT)

## Abstract

**Background:**

Observing medication ingestion through self-recorded videos (video directly observed therapy [VDOT]) has been shown to be a cost-effective alternative to in-person directly observed therapy (DOT) for monitoring adherence to treatment for tuberculosis disease. VDOT could be a useful tool to monitor short-course latent tuberculosis infection (LTBI) treatment.

**Methods:**

We conducted a prospective randomized controlled trial comparing VDOT (intervention) and clinic-based DOT (control) among patients newly diagnosed with LTBI who agreed to a once-weekly 3-month treatment regimen of isoniazid and rifapentine. Study outcomes were treatment completion and patient satisfaction. We also assessed costs. Pre- and posttreatment interviews were conducted.

**Results:**

Between March 2016 and December 2019, 130 participants were assigned to VDOT (n = 68) or DOT (n = 62). Treatment completion (73.5% vs 69.4%, *P* = .70) and satisfaction with treatment monitoring (92.1% vs 86.7%, *P* = .39) were slightly higher in the intervention group than the control group, but neither was statistically significant. VDOT cost less per patient (median, $230; range, $182−$393) vs DOT (median, $312; range, $246−$592) if participants used their own smartphone.

**Conclusions:**

While both groups reported high treatment satisfaction, VDOT was not associated with higher LTBI treatment completion. However, VDOT cost less than DOT. Volunteer bias might have reduced the observed effect since patients opposed to any treatment monitoring could have opted for alternative unobserved regimens. Given similar outcomes and lower cost, VDOT may be useful for treatment monitoring when in-person observation is prohibited or unavailable (eg, during a respiratory disease outbreak). The trial was registered at the National Institutes of Health (ClinicalTrials.gov NTC02641106).

**Clinical Trials Registration:**

ClinicalTrials.gov NTC02641106; registered 24 October 2016.

## Teaser

Latent tuberculosis infection treatment completion was similar between participants monitored by standard-of-care directly observed therapy and asynchronous video directly observed therapy; however, for video directly observed therapy, participants reported higher treatment satisfaction and the cost of treatment monitoring was less.

Over 85% of US tuberculosis infections result from latent tuberculosis infection (LTBI) reactivation [1], making LTBI treatment central for tuberculosis elimination [2]. Treatment with isoniazid regimens has shown statistically significant reductions in reactivation risk [3, 4], yet only 3% of the 10 to 15 million individuals with LTBI in the United States initiate treatment annually [5, 6]. Approved short-course regimens, such as 3 months of isoniazid and rifampicin given in 12 weekly doses (3HP) [7], could increase treatment initiation and completion; however, reluctance to administer it without directly observed therapy (DOT) persists even after the Centers for Disease Control and Prevention relaxed DOT requirements in 2018 [8].

DOT is an established patient-centered alternative to self-administered antituberculosis treatment in which a health worker or designee observes patients ingesting each medication dose in a clinic or community-based setting to increase treatment completion and potentially lower costs [9–11]. However, because other LTBI treatment regimens are self-administered, DOT places burden on patients that lowers adherence (eg, time, lost wages for time off work, transportation). Additionally, patients who live far from treatment centers and have fewer resources (eg, migrants and low-income patients) may not be offered 3HP [12]. Nonetheless, one study that assessed DOT for LTBI treatment via multiple treatment regimens found that among 15 035 patients who initiated treatment, 71.4% using DOT completed treatment as compared with 45.2% overall [13]. Recent studies of DOT examined synchronous/real-time video conferencing (VDOT) to avoid the need for travel by patients or providers, and they showed that it can increase adherence, improve patient satisfaction, and reduce monitoring costs for antituberculosis treatment [14–16]. Regarding LTBI treatment, a nonrandomized trial in New York City found that 3HP treatment completion was higher for synchronous VDOT vs clinic-based DOT (88% vs 65%, *P* < .001) [17]. Yet, internet instability and the need for patients and providers to be simultaneously available were noted limitations of synchronous VDOT [18, 19].

This study evaluated asynchronous VDOT to overcome the limitations of synchronous VDOT. Asynchronous VDOT uses smartphone applications that can store and forward recorded videos made by patients while ingesting their medications online or offline at any time of day, which health care providers subsequently view to document adherence. Asynchronous VDOT has been shown to increase adherence and was associated with greater patient satisfaction and lower cost than DOT for antituberculosis treatment [16, 20–22]; however, no studies have examined whether asynchronous VDOT improves 3HP treatment completion as compared with DOT. This study evaluated whether asynchronous VDOT is associated with higher treatment completion, greater satisfaction, and lower costs than clinic-based, in-person DOT for LTBI treatment with 3HP.

## METHODS

### Study Design

This multisite study employed a parallel-arm randomized controlled trial design with participants allocated to VDOT (intervention) or standard-of-care clinic-based DOT (control). Participants were interviewed prior to randomization (baseline) and again at the end of the study (follow-up) to assess their characteristics, treatment satisfaction, and cost of receiving care. This study was approved by the Human Research Protections Program of the University of California San Diego and the Division of Microbiology and Infectious Diseases of the National Institutes of Health.

### Participants and Setting

Eligible participants were qualified for 3HP [7], at least 13 years old, not planning to move out of San Diego County within 4 months of study enrollment, and willing and able to participate and provide informed consent. Individuals were excluded if they were unwilling to allow access to their medical records, incarcerated, enrolled in a court-ordered alcohol/drug treatment program, or were physically unable (eg, blind) or cognitively unable to use VDOT, unless a household member could assist them throughout the study. Ten months after recruitment began, the protocol was revised to include individuals in voluntary substance use treatment programs.

Participants were recruited from 7 San Diego County TB Control and Refugee Health Program clinics and the University of California San Diego’s Student Health Services clinic. All sites screened high-risk populations (eg, foreign born, tuberculosis contacts, travelers to endemic areas, and persons with HIV).

### Patient Consent Statement

Adult participants provided written informed consent. Minors (age, 13–17 years) provided written assent in addition to written parent/guardian consent. This study was approved by the University of California San Diego Institutional Review Board (150155).

### Screening and Enrollment

Patients with diagnosed LTBI were offered treatment with 9 months of daily self-administered isoniazid, 4 months of daily self-administered rifampin (4R), or 3HP. Patients who selected 3HP were invited to learn about the study and enroll if interested. Patients who refused participation received usual care, which could include 3HP with clinic-based DOT.

### Intervention and Control Conditions

#### VDOT Arm: Intervention.

VDOT participants were instructed to record themselves ingesting each weekly medication dose, and clinic staff documented their observations (eg, number of pills swallowed, patient comments, video/audio quality). Participants were loaned a smartphone equipped with the VDOT app and cellular service. The first dose was taken with a clinic staff member present during VDOT app training. The app automatically date- and time-stamped, encrypted, and uploaded each video to a secure server, which prevented tampering by participants.

#### In-person DOT Arm: Control.

Participants assigned to DOT took their first dose in person with clinic staff watching and were scheduled to return for weekly observed dosing until treatment completion.

### Randomization

Following eligibility screening, informed consent, and baseline interview, participants were randomly assigned 1:1 to trial arms with variable block sizes of 4 and 6. Study personnel used a web-based randomization service (Sealed Envelope) to determine treatment assignment. In 3 instances, 2 participants from the same household were enrolled in the study. To avoid intervention contamination, it was decided a priori to randomize 1 participant per household and administratively assign all other eligible household members to the same trial arm.

### Measures and Outcomes

#### Participant Interviews.

Participants completed a brief baseline interview (15–20 minutes) to assess sociodemographics, smartphone ownership, mode of transportation for clinic visits, recent travel, homelessness, substance use, tuberculosis knowledge and attitudes, and perceptions of LTBI treatment monitoring. A follow-up interview assessed satisfaction with and perceptions of treatment observation and clinical care, medication taking patterns, out-of-pocket treatment costs, and VDOT experience (VDOT arm only). The interviews were conducted by research staff, rather than clinic staff, to minimize socially desirable responding.

#### Medical Record and Treatment Log Abstraction.

LTBI diagnosis, treatment adherence, drug side effects, and final treatment disposition were abstracted from patient medical records. In addition to dose information captured in the VDOT provider dashboard, clinical staff maintained standardized paper treatment logs. Treatment log data were abstracted and compared with the electronic records. Discrepancies occurred infrequently and were reconciled by clinical staff members.

#### Cost Data Collection.

Field observations and interviews with providers were used to assess provider costs (Supplementary material). We divided treatment delivery into mutually exclusive clinical and administrative tasks (eg, laboratory processing, in-person contact, charting). Onetime tasks (eg, LTBI chart setup) and recurrent tasks (eg, entering dose observations) were documented. VDOT-specific tasks included registering participants in the VDOT platform, watching videos, and closing out participant records upon treatment completion. We assumed that a basic smartphone could be used over 4 treatment courses per year for 3 years with a corporate price of $150. We estimated mobile phone service to cost $25 per month based on government rates. Cost of the VDOT app was estimated at $12 for a full course of LTBI treatment based on commercial subscription pricing in the United States. Participant LTBI treatment costs included self-reported time off work, time spent traveling round trip to the clinic, and transportation.

#### Primary Outcome.

The primary outcome measure was treatment completion (ie, ingested 12 doses within 16 weeks). Only doses observed in person or by video were counted as taken. Participants who stopped treatment early or switched to a different regimen were considered incomplete. Participants had a 3-day window before and after each scheduled dose date to be counted in that week. Doses taken outside the window were counted toward the following week, and the treatment schedule was extended 1 week for each missed dose.

#### Secondary Outcomes.

To see if VDOT promoted more timely completion, we assessed completion as taking 12 doses within 11 weeks of the first dose. Since providers may consider 11 doses within 16 weeks as complete treatment [23] and less effort might be made to ensure that the 12th dose was taken, we also conducted an analysis defining completion as taking 11 doses within 16 weeks.

We further examined the adherence rate by calculating the number of doses observed divided by the number of times that a dose was expected. Missing a weekly dose added to the number of expected observations. For this analysis, if participants stopped treatment early or switched to a different regimen, the number of doses expected was considered the number of weeks in the study before stopping treatment.

### Sample Size and Statistical Analysis

**Figure 1.**
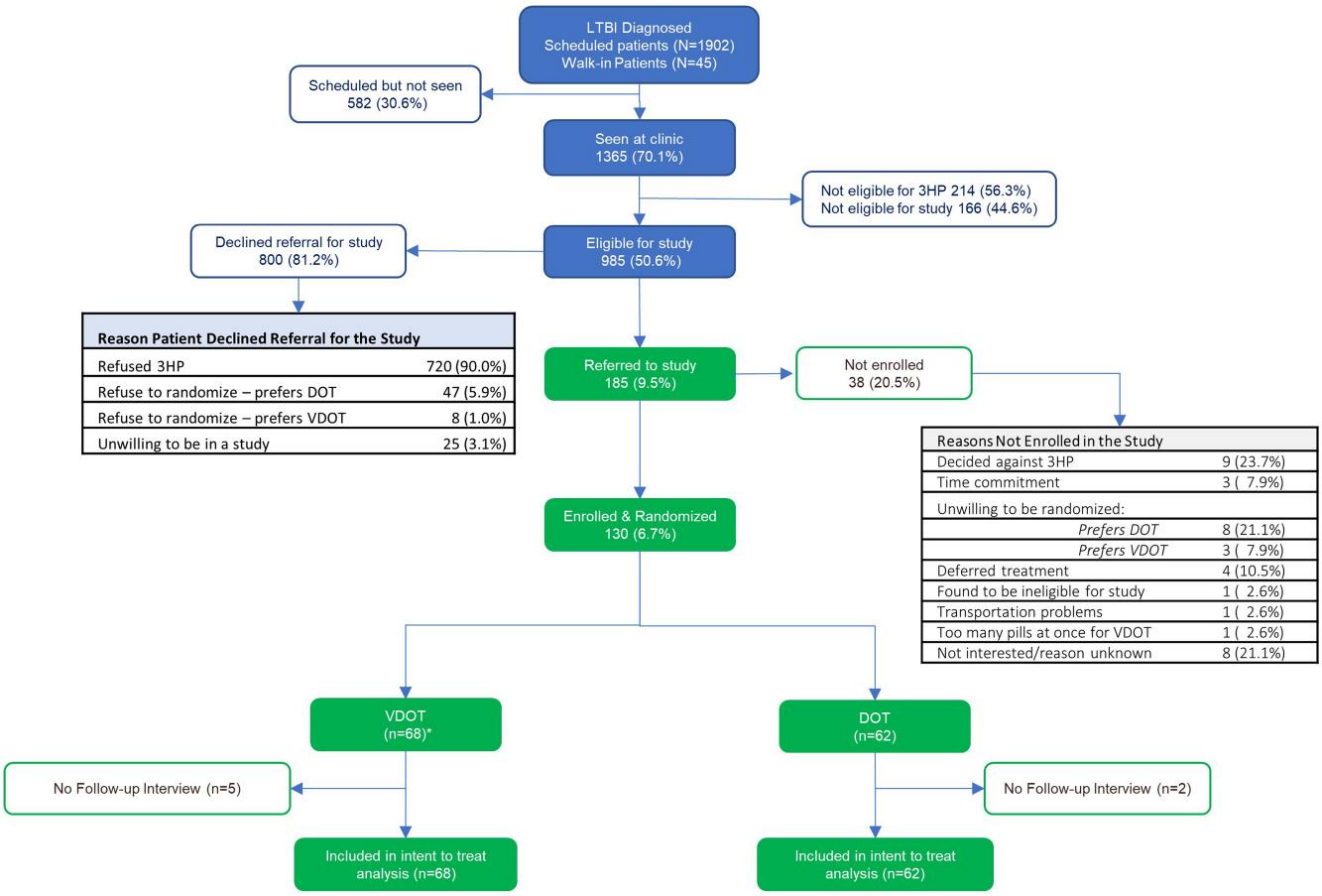
Participant flow diagram (March 2016–March 2020). Individuals with LTBI who were designated by a health care provider to be eligible for LTBI treatment with the 3-month weekly isoniazid/rifapentine (3HP) regimen were referred to study staff for recruitment. Those who agreed and provided written informed consent were randomly assigned to the intervention group (VDOT) or standard-of-care control group (DOT), with the following exceptions: the first 5 participants, who were administratively assigned to the VDOT arm to give clinical staff early experience using the intervention following training, and the last participant, who could only be assigned to VDOT due to COVID-19 restrictions on in-person clinic visits. All participants had data on treatment completion and adherence; however, 5 VDOT and 2 DOT participants did not complete the follow-up interview and have missing data on treatment experience. DOT, directly observed therapy; LTBI, latent tuberculosis infection; VDOT, video directly observed therapy.

Sample size was based on comparing the proportion of patients completing treatment between VDOT and DOT. Assuming a 2-sided test with an alpha of .05, an expected completion of 75% for DOT and 90% for VDOT, and 80% power to detect this difference, we determined that a sample size of 113 per group was needed. We also considered the cluster effect of enrolling multiple participants from the same household, given a mean 1.5 patients per household receiving LTBI treatment in San Diego County (personal communication, Maria Luisa Moore, 23 May 2013). This increased the target sample size to 155 participants per arm. Due to enrollment delays, we did not achieve the target sample size. Therefore, we calculated actual study power according to the sample size obtained (Figure 1). With the original parameters, treatment completion in the VDOT arm would have to be at least 92.5% (absolute 17.5% increase) to detect a statistically significant difference between arms. Sample size was calculated by PASS software (2008) and R software [24].

Wilcoxon rank sum tests and chi-square tests (or Fisher exact tests as appropriate) were used to investigate differences in participant characteristics by trial arm and determine the need to control for confounding. The primary analysis used a modified intent-to-treat approach that included all randomized participants. All participants had complete outcome data; 5 VDOT participants and 2 DOT participants were missing follow-up interview data. Fisher exact tests and simple logistic regression were used to assess the difference in proportion of treatment completion between groups. Given the small number of household clusters (3 households had 2 participants each), we ignored the cluster effect in the primary analysis and performed a sensitivity analysis by removing these 6 participants. All analyses were performed with 2-sided tests with an alpha of .05. Blinding participants and staff to treatment assignment was impossible; however, the statistician conducting the analysis was blinded throughout the trial. All analyses were performed with R software [24].

## RESULTS

### Study Population

Enrollment occurred between March 2016 and March 2020, during which >1900 individuals were diagnosed with LTBI by the study clinics, of which 30% (n = 582) did not return to be offered treatment (Figure 1). An additional 20% were either ineligible for 3HP (n = 214) or did not meet other study criteria (n = 166). Of the 985 patients eligible for the study, 81% declined to hear about it—the main reason being disinterest in 3HP, most of whom opted for self-administered 4R (Supplementary Table 1). Recruitment ended before reaching the target sample size due to COVID-19–related clinic restrictions. Follow-up ended in June 2020.

Overall, 185 patients were referred to the study and 130 enrolled (Figure 1). There were no statistically significant differences in baseline participant characteristics by trial arm (Table 1). Both arms included minors and 95% of participants currently owned a smartphone. Participants spent a half hour on average traveling one-way to the clinic, and most considered LTBI “somewhat serious” or “very serious.” At baseline, <5% of participants said treatment monitoring made them feel “patronized,” “not trustworthy,” or “embarrassed,” while most (79%) said that they “don’t mind” having someone watch them take LTBI medications.

**Table 1.** Baseline Characteristics of VDOT for Monitoring Adherence for LTBI Treatment by Trial Arm, 2016–2020 (N = 130)

	No. (%) or Median (IQR; Range)	
Characteristic	Intervention (n = 68)	Control (n = 62)
Clinic		
County tuberculosis program	57 (84)^a^	53 (85)
University of California San Diego, student health	11 (16)	9 (15)
Time traveling 1 way to clinic, min	20 (15–30; 5–120)	20 (15–30; 5–150)
Age, y	33 (23–42.3; 13–75)	37 (23–42.8; 14–57)
Sex		
Male	25 (37)	26 (42)
Female	43 (63)	36 (58)
Race/ethnicity		
Latinx/Hispanic	46 (68)	40 (65)
Asian	18 (27)	13 (21)
Black, non-Hispanic	2 (3)	4 (7)
White, non-Hispanic, or mixed race	2 (3)	5 (8)
Primary language		
English	18 (27)	15 (24)
Spanish	35 (52)	33 (53)
Other language^b^	15 (22)	14 (23)
Country of birth		
US	16 (24)	14 (23)
Mexico	28 (41)	27 (44)
Other country	24 (35)	21 (34)
Came to the US as a refugee		
Yes	6 (12)	2 (4)
No	45 (88)	46 (96)
Highest grade completed		
Middle school or lower: ≤8 y	6 (9)	8 (13)
High school: 9–12 y	25 (37)	19 (31)
Some college	21 (31)	18 (29)
Bachelor degree or higher	16 (24)	17 (27)
Personal income: past 12 mo, $		
<10 000	38 (58)	34 (55)
10 000−30 000	19 (29)	21 (34)
>30 000	9 (14)	7 (11)
Employed full-time outside of home		
Yes	18 (27)	13 (21)
No	50 (73)	49 (79)
Own a smartphone		
Yes	64 (94)	60 (97)
No	4 (6)^c^	2 (3)
Alcohol use: past 1 mo		
Never	31 (46)	28 (45)
<1/mo	2 (3)	6 (10)
1–3 d/m	21 (31)	15 (24)
≥1/wk	14 (21)	13 (21)
Smoked marijuana: past 6 mo		
Yes	8 (12)	7 (11)
No	60 (88)	55 (89)
Ever incarcerated		
Yes	4 (6)	5 (8)
No	64 (94)	57 (92)
Times traveled out of county: past 6 mo		
Never	21 (31)	19 (31)
<1/mo	23 (34)	22 (36)
1–3 d/mo	10 (15)	7 (12)
≥1/wk	13 (19)	13 (21)
Perceived treatment monitoring as patronizing, untrusted, or embarrassing		
Yes	2 (3)	4 (7)
No	66 (97)	58 (93)
Don’t mind being watched taking medications		
Yes	53 (78)	49 (79)
No	15 (22)	13 (21)
Perceived seriousness of LTBI		
Very serious	26 (39)	24 (39)
Somewhat serious	32 (49)	27 (44)
Not at all serious	8 (12)	11 (18)

Abbreviations: IQR, interquartile range; LTBI, latent tuberculosis infection; VDOT, video directly observed therapy.

^a^Includes 1 participant enrolled through a noncounty federally qualified health center.

^b^Other languages included Tagalog, Tamil, Vietnamese, Cantonese, Arabic, Hmong, Portuguese, Korean, Lingala, and Khmer.

^c^Includes individuals who do not own any type of cellular phone.

### Treatment Completion and Adherence

**Figure 2.**
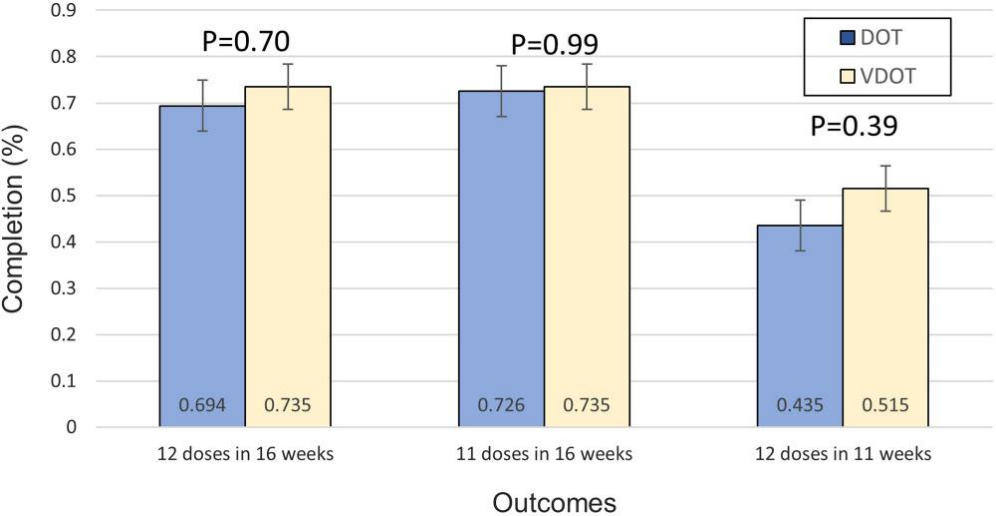
Treatment completion rates by trial arm. Dark bars represent the intervention arm (VDOT, n = 68), and light bars represent the control arm (DOT, n = 62). The primary aim is ingestion of 12 doses within 16 weeks of treatment initiation. The secondary outcomes included ingestion of 11 doses within 16 weeks of treatment initiation (accepted as completion by providers) and ingestion of 12 doses within 11 weeks of treatment initiation (completion on schedule with no missed doses). Error bars indicate 95% confidence interval. DOT, directly observed therapy; VDOT, video directly observed therapy.

Over two-thirds of participants in both arms completed treatment (Figure 2). Completion was higher in the VDOT arm for the primary outcome (12 doses taken within 16 weeks) and secondary outcomes (12 doses taken within 11 weeks and 11 doses taken within 16 weeks); however, these differences were not statistically significant (Table 3). There was also no difference in mean treatment adherence (ie, doses taken on schedule) between VDOT and DOT (87.2% vs 88.1%, *P* = .32).

### Participant Experience and Intervention Acceptance

We examined whether the trial arm affected whether participants ate before each dose since food helps to alleviate some drug side effects [25]. Over two-thirds of participants reported eating a meal or snack, which did not differ between DOT (72%) and VDOT (70%). Treatment monitoring had little or no impact on daily life for most participants and did not vary by trial arm. More reported sharing LTBI treatment experiences with family members rather than friends, neighbors, schoolmates, or coworkers (Table 2).

**Table 2.** Participant Self-reported LTBI Treatment Experience and Acceptance at Follow-up by Trial Arm, 2016–2020

	Participants, No. (%)		
Characteristic	Intervention (n = 63)	Control (n = 60)	*P* Value
Shared LTBI treatment experience with family members?			.24
Yes	49 (78)	52 (87)	
No	14 (22)	8 (13)	
Shared LTBI treatment experience with friends, neighbors, schoolmates, coworkers?			.59
Yes	35 (56)	30 (50)	
No	28 (44)	30 (50)	
Traveled outside the county during treatment period?			.37
Yes	39 (62)	32 (53)	
No	24 (38)	28 (47)	
Ate meal or snack before taking medications?			>.99
Yes	43 (72)	42 (70)	
No/no pattern	17 (28)	18 (30)	
Impact of treatment monitoring on daily life: 1 = no impact, 10 = major impact)			.56
1	48 (76)	42 (70)	
2–4	8 (13)	7 (12)	
5–10	7 (11)	11 (18)	
Satisfaction with clinical care overall			.49
Neutral or very dissatisfied	0 (0)	1 (2)	
Somewhat or very satisfied	63 (100)	59 (98)	
Satisfaction with method of adherence monitoring			.39
Neutral or very dissatisfied	5 (8)	8 (13)	
Somewhat or very satisfied	58 (92)	52 (87)	
Preferred method if had to redo treatment			<.001
Video directly observed therapy	58 (92)	30 (50)	
Directly observed therapy	1 (2)	17 (28)	
No preference	4 (6)	13 (22)	
Would recommend my treatment method for others			<.001
No	1 (2)^a^	10 (17)	
Yes	62 (98)	50 (83)	

^a^One participant in the intervention arm who responded “don’t know” was coded as “no” for analysis.

Regarding treatment satisfaction, nearly all were “somewhat or very satisfied” with their clinical care, and most were “somewhat or very satisfied” with their monitoring method; differences by trial arm were not statistically significant (Table 2). Notably, while 92% of VDOT participants would choose VDOT if they had to redo their treatment, 50% in the DOT arm said that they would prefer VDOT despite never having used it (*P* < .001). While most participants would recommend their treatment monitoring method to other patients with LTBI, a larger proportion favored VDOT (*P* < .001).

### Cost by Trial Arm

The human resources needed to administer VDOT and DOT and the participant out-of-pocket expenses were assessed at each site (Supplementary Table 2) and used to compute treatment costs (Table 4). The median total personnel cost per patient was 10% less for VDOT ($204) than for DOT ($229). The median combined patient and provider costs per patient for a 12-week course of VDOT and DOT were $318 (range, $270–$480) and $312 (range, $246–$592), respectively. However, if participants had the option of using their own smartphones and mobile phone services, VDOT ($230; range, $182–$393) would cost 26% less than DOT.

**Table 3.** Absolute Risk and Relative Risk for Treatment Adherence

	Participants, No. (%)			
	VDOT (n = 68)	DOT (n = 62)	Risk Difference (95% CI)^a^	Odds Ratio (95% CI)^b^
Primary outcome				
Treatment completion per guidelines	50 (73.5)	43 (69.4)	4.1 (−12.9, 21.2)	1.23 (.57–2.63)
Secondary outcomes				
Completing 11 doses within 16 wk	50 (73.5)	45 (72.6)	0.9 (−15.3, 17.2)	1.05 (.48–2.28)
Completing 12 doses within 11 wk	35 (51.5)	27 (43.5)	8.0 (−10.7, 26.6)	1.38 (.69–2.75)

Abbreviations: DOT, directly observed therapy; VDOT, video directly observed therapy.

^a^95% CI for difference is based on 2 proportion tests with continuity correction.

^b^Reference group is the DOT arm.

**Table 4.** LTBI Treatment Monitoring Costs by Trial Arm

	Median (Range) , $	
	Intervention	Control
Personnel	204 (163–341)	229 (206–352)
Patient costs	14 (7–40)	83 (40–240)
Device charge^a^	88	0
VDOT app charge	12	…
Total	318 (270–480)	312 (246–592)
Total: excludes device charge	230 (182–393)	312 (246–592)

Abbreviations: LTBI, latent tuberculosis infection; VDOT, video directly observed therapy.

^a^Device charges include smartphone and cellular service provided by the study, which may be excluded if patients use their own smartphones.

## DISCUSSION

In this trial, we did not observe a statistically significant difference in treatment completion or adherence between the intervention (VDOT) and control (DOT) arms, although point estimates suggest better outcomes in the VDOT arm. Satisfaction with clinical care and treatment adherence monitoring were very high overall and did not differ by trial arm; however, more participants in both arms preferred VDOT over DOT, and a greater proportion of VDOT participants than DOT participants would recommend their monitoring method for other patients receiving 3HP. The combined patient and provider cost to administer a 12-week course of 3HP was similar between arms, but VDOT was estimated to have lower costs than DOT if patients used their own smartphones and data plans.

Overall treatment completion rates were lower in this study than in other studies of 3HP. A study reporting adherence by DOT vs self-administration in the United States [26] found a slightly higher completion rate among patients using DOT (77.9%) as compared with participants in the DOT arm of our study (72.6%). This difference between the studies could be due to variability in DOT administration, patient characteristics, and difficulty traveling to the clinic.

Although we observed no difference in LTBI treatment adherence, like that reported from antituberculosis treatment trials [20, 27], we did observe a similar preference for VDOT over DOT. Feasibility and acceptability studies of VDOT for LTBI have also reported high acceptance and treatment completion. For example, among 16 pediatric patients using synchronous VDOT for 3HP, 100% completed treatment, which saved patients time by avoiding an average of 51 minutes of travel time one-way to the clinic [28].

Participants in this study were loaned a smartphone with a data plan to standardize VDOT app training and avoid potential service interruptions. Since 95% of participants owned a smartphone prior to the study, it is reasonable to expect that most patients could use VDOT on their own smartphones, making VDOT cost saving over DOT; however, some accommodation might be necessary for any patient whose smartphone or operating system is incompatible with the chosen VDOT app. Also, because reported income was low overall, the self-reported loss of income due to attending in-person clinic visits in our study contributed little to the cost of LTBI treatment monitoring; thus, patients with higher incomes might achieve greater cost savings from VDOT.

### Limitations

Several limitations could have affected our findings. The final sample size was smaller than proposed because most eligible patients opted for the self-administered 4R regimen that was introduced at the time the trial started. Anecdotal reports from health care providers suggest 2 main reasons for patient preferences: (1) self-administered 4R, despite longer duration, was preferred over weekly DOT clinic visits, and (2) 3HP had a larger pill burden (9 vs 2 tablets per dose). Another factor affecting enrollment was a rifapentine shortage that interrupted initiation of 3HP during the study. In addition, changes in federal immigration law [29] during the trial greatly reduced tuberculosis screening at the county refugee health clinic where we expected to recruit many participants. Volunteer bias potentially dampened the effect of VDOT on treatment completion. Since study participants had to accept their assigned arms, patients who knew that they would have difficulty attending weekly clinic visits for DOT (eg, missing work) would have opted out. Consequently, oversampling individuals capable of in-person DOT could have biased our findings, making treatment completion and adherence similar across trial arms. Full-time employment was low among study participants (24%), which supports the hypothesis that volunteers had fewer barriers to attending in-person DOT visits.

In conclusion, we did not observe a statistically significant increase in LTBI treatment completion among patients using VDOT vs in-person DOT. Preference for VDOT was greater than DOT in both arms, suggesting that patient satisfaction could be increased by offering VDOT as an option for LTBI treatment monitoring. Also, VDOT was cheaper than DOT if patients used their own smartphones. Notably, uptake of 3HP in San Diego County was low overall, potentially due to the DOT requirement and high pill burden. Further research is needed to understand whether uptake and completion of the 3HP regimen would increase if offered as self-administered treatment and if these measures differ as compared with administration by VDOT.

## Supplementary Material

ofae180_Supplementary_Data

## Supplementary Data

Supplementary materials are available at *Open Forum Infectious Diseases* online. Consisting of data provided by the authors to benefit the reader, the posted materials are not copyedited and are the sole responsibility of the authors, so questions or comments should be addressed to the corresponding author.

## Notes

***Acknowledgments.*** We thank the study participants for agreeing to take part in this research. We are also grateful for the cooperation and invaluable feedback provided by the participating clinics. Patricia Marin was instrumental in programming and maintaining the VDOT platform for this study at the University of California San Diego.

***Author contributions*.** Conception, design, or planning of the study: R. S. G., L. L., J. C., J. C.-M., C. A. B. Acquisition of data: S. G., S. S. M., A. A., J. C.-M., V. M., M. M., P. R., F. R. Analysis of data: L. L., J. C., D. G. C., P. R., F. R. Interpretation of data: R. S. G., L. L., S. G., S. S. M., J. C.-M., D. G. C., C. A. B. Drafting of the manuscript: R. S. G., L. L., J. C., J. C.-M., C. A. B. All authors critically reviewed or revised the manuscript for important intellectual content and approved the final version.

***Data availability.*** Data are available from the corresponding author.

***Disclaimer*.** The funder reviewed and approved the study protocol but had no role in study implementation, data analysis, data interpretation, or preparation of this manuscript.

***Financial support*.** This work was supported by the National Institute of Allergy and Infectious Diseases at the National Institutes of Health (grant #U01-AI116392).
