# Supplementary material for: Asynchronous Video Directly Observed Therapy to Monitor Short-Course Latent Tuberculosis Infection Treatment: Results of a Randomized Controlled Trial
Source: Open Forum Infect Dis. 2024 Mar 26;11(4):ofae180. doi: 10.1093/ofid/ofae180 (PMC11045025; doi:10.1093/ofid/ofae180)
Supplement: ofae180_Supplementary_Data [file ofae180_supplementary_data.docx]

**Supplement to:** Asynchronous Video Directly Observed Therapy (VDOT) to Monitor Short-Course LTBI Treatment: Results of a Randomized Controlled Trial

**Contents:**

- Supplemental Methods
  - VDOT Intervention Description
  - Medication Reminders
  - Monitoring Medication Side Effects
  - Participant Retention
- Supplemental Results
  - Recruitment Outcomes
  - Medication Side Effects
- Supplemental Cost Analysis Methods and Results
- Table S1 - Reasons Given by Patients for Refusing to Participate in the Study
- Table S2 - Percent Effort and Number of Minutes for Personnel to Complete VDOT and DOT Related Tasks by Site
- Table S3 - Cost Breakdown for Delivery of 3HP Regimen for LTBI Treatment Using VDOT or DOT
- Figure S1 - Median Lower and Upper Bounds of Minutes Spent Viewing Videos Based on Data Captured in the VDOT Patient Management System

**Supplemental Methods:**

*VDOT Intervention Description:* Medications were dispensed four doses at a time, and patients were instructed to take them at the same day/time each week. Research personnel taught participants how to use the smartphone and VDOT app, record videos (i.e., remain in-frame, show each tablet placed in mouth and swallowed), and troubleshoot problems using the app. Parents/guardians of minors in the VDOT arm were also taught to use the app so they could assist if necessary. Participants received a pamphlet containing VDOT app instructions for reference. VDOT participants practiced recording videos until they mastered the process, and then recorded themselves taking their first medication dose with clinic staff present. The remaining doses were recorded independently at the participant’s chosen location. Additional training was provided by clinic staff if participants had problems with the smartphone, VDOT application, and/or recording procedures.

*Medication Reminders:* Participants in both arms received weekly medication reminders via email or text message, unless they opted out. VDOT arm participants received automatic messages when each video was due. A second message was sent if the video was not received within one hour of the first message. Control arm participants received a reminder message 24 hours before their weekly clinic appointment. If participants in the control arm failed to attend scheduled appointments, clinic staff called them to encourage adherence and reschedule appointments. Clinic staff received daily reports listing the ID numbers of VDOT participants whose videos were missing. These participants were contacted to determine whether the medication was taken and troubleshoot potential problems. Throughout treatment, clinic staff followed established clinic protocols for contacting patients who missed doses in both trial arms.

*Monitoring Medication Side Effects:* A potential unintended consequence of asynchronous VDOT use is that drug side effects common to 3HP might go undetected among patients who are not seen in person. For example, 362 (62.5%) participants in one study experienced one or more side effect, of which 38 (10.5%) and 98 (27.1%) required temporary and permanent treatment interruption, respectively.^30^ Therefore, participants in our study were evaluated for drug side effects by a healthcare provider during monthly clinic visits. Signs and symptoms of drug toxicity were documented at each visit, and laboratory tests were ordered as needed. All participants were scheduled to return at weeks four and eight. A 12-week visit was added if treatment took more than 12 weeks to complete. Participants were instructed to call their provider if side effects occurred between visits and were also documented. Participants were also asked during the follow-up interview if they changed their LTBI treatment regimen after enrolling in the study, and if they did, what were their reasons for changing their regimen. Stopping treatment was included with changing regimens.

*Participant Retention:* To minimize attrition, we collected comprehensive contact information at enrollment. Clinic staff updated contact information if it changed during the study and assisted research staff when participants could not be reached for follow-up interviews. Participants were compensated $25 per interview ($50 total) in the form of a check or gift card for their time. No compensation was given for treatment adherence, attending clinic visits, or sending VDOT videos.

**Supplemental Results:**

*Recruitment Outcomes:* Of the 800 patients that provided reasons for declining referral to the study in Figure 1, most refused 3HP because of scheduling and transportation concerns related to DOT (Table S1). Some patients refused the study because they preferred either DOT or VDOT and would not be willing to continue the study if randomized to the alternate arm. Nineteen patients reported either refusing 3HP or preferring to take the large number of pills (9 tablets per dose) with a provider present. LTBI treatment providers reported that most patients opted for the self-administered 4R regimen instead of 3HP.

Overall, 185 patients were referred to the study and 130 were enrolled (Figure 1). All participants were randomly assigned to treatment arms, except the first five who were administratively assigned to the VDOT arm to minimize the interval between staff training and first VDOT use, and the last participant who could only receive VDOT due to COVID-19 in-person clinic restrictions. All participants had complete treatment data; five participants in the VDOT arm and two in the DOT arm did not complete the follow-up interview.

*Medication Side Effects:* The proportion of participants reporting side effects was similar in the VDOT (54%) and DOT (52%) arms (p=0.86). Of those reporting any side effects, 64% reported the occurrence following one or two doses; only one participant reported side effects after every dose (data not shown). Overall, 23/113 (20.4%) of participants changed or stopped LTBI treatment regimens during the study, of whom all but one did so because of medication side effects, which did not differ by trial arm (100% in VDOT vs. 91.7% in DOT).

**Supplemental Cost Analysis Methods and Results:**

1. **Methods**

*Data Collection:* All data were collected from March 2016 to June 2020. Based on field observations and interviews with providers, we divided treatment delivery into mutually exclusive clinical and administrative tasks (e.g., laboratory processing, in-person contact, charting). Frequency of some tasks varied, with some tasks completed once throughout the entire treatment course (e.g., LTBI chart set up) while other tasks were recurrent (dose observation notes). VDOT-specific tasks included registering participants on the VDOT platform, watching videos, and closing out participant records upon treatment completion. We excluded effort and costs related to missed appointments as they were infrequent.

Participants in both arms received automated text message reminders. Those in the DOT arm received reminders one day prior to their clinic appointment and those in the VDOT arm received reminders one hour prior to when their dose was due. Only one clinic made phone call reminders to each participant prior to their appointments because it was already part of that clinic’s standard protocol. Since phone call reminders were not conducted in other clinics, we did not include the cost of tasks for non-automated dosage reminders.

To obtain the cost of adherence monitoring, we administered questionnaires to clinic providers involved in the study and patients participating in the trial. With the provider questionnaires, we elicited information on the personnel completing each task, percent effort, and the mode, minimum, and maximum number of minutes required to complete each task per participant. The mode, minimum, and maximum minutes per task were not calculated through direct observation but self-reported by the provider. Providers’ self-reported time viewing videos was triangulated with meta-data collected as part of the Client Management System (CMS). The CMS is the online platform used by the VDOT system to view videos, document doses taken/missed, and monitor treatment adherence. We calculated the length of videos viewed based on CMS data by using the start and end timestamp embedded in meta-data for each video.

We assigned a fair-market price for a smartphone with the minimum requirements to run the VDOT software to cost approximately $150 and have a lifespan of three years. We assumed the smartphone could be used over four treatment courses, per year. Based on government rates, we estimated mobile phone service to cost approximately $25 per month. The cost of the VDOT application was set at $12 for a full course of treatment based on rates provided by a commercial VDOT service provider.

*Costing Methodology:* We adopted a societal perspective where all costs were estimated to reflect the real cost of the resources (irrespective of whether they were purchased or donated). We used an ingredients-based micro-costing approach to estimate the 12-week treatment delivery cost for each arm. Since the focus of the analysis was on estimating the delivery of treatment by modality, we did not include the cost of 3HP medication, laboratory, or other diagnostic tests, which were equivalent in both arms.

Treatment delivery tasks could have been completed by different clinical personnel (registered nurse, public health nurse, licensed vocational nurses), thus the cost per task was weighted by the percent effort and salary of the individual completing the task. As most providers were employed at public institutions, we obtained the yearly salary, fringe benefits, and other compensation, from a publicly available website that reports yearly compensation of state employees.^1^ We converted yearly salaries to a per minute wage, assuming full time employment and a 40-hour workweek. If we could not find the salary for a specific staff member, then we assumed it was equivalent to known salaries based on a similar position.

We estimated participant costs on self-reported measures obtained from the baseline and follow-up questionnaires. We elicited data on the time taken off work, time spent traveling round trip to the clinic, transport costs (among those who reported using public transportation), and fuel costs (among those who used a car as their primary mode of transport to the clinic). To calculate the cost associated with time off from work, we estimated the hourly wage based on self-reported monthly income and number of hours worked per month. For individuals who reported working but not receiving an income, we assumed an hourly wage of $12/hour (minimum wage in California in 2020). Further, we assumed that individuals who reported receiving a monthly income but did not report any hours, worked part-time (20 hours per week). We estimated the fuel cost to be $0.17 per mile, the standard mileage rate for medical purposes.^2^ The participant cost was the sum of the costs associated with time off from work, travel time to the clinic, out-of-pocket transportation costs, and fuel costs.

*Outcomes and Statistical Analyses:* Our primary outcome was the per person adherence monitoring cost of completing 3HP treatment for those receiving either VDOT or DOT. We assumed a full treatment course consisting of 12, once weekly in-person visits for DOT and 12 video recordings and once monthly in-person DOT visits for those receiving VDOT (patients on VDOT visited the clinic monthly). We also considered the cost of VDOT provision excluding smartphone-related costs (e.g., phone, cellular service, app). Provider and participant measures were analyzed using SAS version 9.4.^3^

1. **Results**

*Personnel effort:* The personnel effort for each task related to VDOT or DOT across the six sites is shown in Table S2. All DOT and VDOT tasks were completed by nursing staff at the county clinics, while a medical resident and field coordinator completed the tasks at the university student health services clinic. A single nurse practitioner was responsible for overseeing service delivery across all county clinic sites. Based on self-reported provider data, the mode number of minutes across the sites for DOT visits ranged from 5 to 25 minutes while the amount of time spent watching each video for patients in the VDOT arm ranged from 2 to 15 minutes. Nearly one-quarter of personnel costs for VDOT were related to VDOT-specific tasks, such as registering participants in the VDOT system, watching videos, and closing-out VDOT records at the end of treatment. Similarly, personnel costs for DOT included charting, appointment scheduling, and observing doses in person.

*Overall costs:* The median total cost of VDOT and DOT over a 12-week treatment course was $318 (range: $270 - $480) and $312 (range: $246 - $592), respectively (Table S3). Additional VDOT-specific costs included loaned smartphone, cellular data plan, and app use fee, which amounted to approximately 31% of the total VDOT delivery cost. If patients had been willing to supply their own smartphone and mobile phone service, then the only additional VDOT related cost would be the application itself ($12 for a full course of treatment), resulting in substantially lower costs $230 ($182 - $393). Most of the cost for both treatment modalities was personnel, which was approximately 64% of the cost for VDOT and 73% for DOT. Nearly one-quarter of personnel costs for VDOT were related to VDOT-specific tasks, such as registering patients in the VDOT system, watching videos, and closing out the patient’s VDOT record. Since VDOT set up and close out were only conducted once per treatment course, the cost for this task was relatively low ($4-$7 per task). However, observation of VDOT videos was more frequent (12 times over the treatment course), resulting in a median cost of $84 (range: $58-$133). There was some variability across the self-reported provider data. As shown in Table S2, most sites reported spending a median of 5 (range 5-10) minutes watching videos, with one site reporting the mode number of minutes watching videos to be 15 minutes (min: 10, max: 20 minutes). However, based on the CMS video data, we did not detect any substantial differences across sites. Thus, we reasoned that the time estimate provided by this site was an outlier and was excluded (Figure S1).

*Participant costs:* Participant costs, consisting of transportation, time off work, and dependent care to attend clinic visits, were 83% lower for VDOT (median=$14) compared to DOT (median=$83). Participants reported a median monthly income and hourly wage of $1,000 (IQR: $0-$2,000) and $10 (IQR: $0-$16) per hour, respectively. Approximately one-third of participants reported no monthly income (n=37, 29%); however, among these individuals, 5 worked more than zero hours per month. Nearly 40% reported working no hours per month (n=52) of which 20 reported a monthly income greater than zero dollars. One-quarter of the sample (n=32) reported no monthly income and no hours worked per month. In terms of loss of productivity, only 28 participants (20%) reported taking time off work for clinic visits, which ranged from 1 to 2 hours per visit at a median cost of $9 per visit. Among individuals who reported taking public transport (n=32), the median cost per trip was $5 (range: $0-$5.50). Further, among those who used either their own car or obtained a ride from a friend/family member, the median round-trip distance was 10 miles (IQR: 5-20 miles) and the median number of hours of roundtrip travel was 0.67 hours (IQR: 0.5-1). Over the entire treatment course, this amounted to a median cost of $30 (IQR: $20-$41). In total, the round-trip cost per person for each in-person clinic visit was approximately $7 (IQR: $3-$20).

**References**

1. Transparent California: California’s Largest Public Pay and Pension Database. 2020; Available at: <https://transparentcalifornia.com/>. Accessed September 13, 2023.

2. Internal Revenue Service. IRS issues standard mileage rates for 2020. Available at: <https://www.irs.gov/newsroom/irs-issues-standard-mileage-rates-for-2020>. Accessed September 13, 2023.

3. SAS Institute Inc. 2016. SAS® 9.4 Language Reference: Concepts, Sixth Edition. Cary, NC: SAS Institute Inc.

| **Table S1. Reasons Given by Patients for Refusing to Participate in the Study (N=800)** | | | |
| --- | --- | --- | --- |
| **Refused 3HP** | | | **720 (90.0%)** |
| Scheduling of DOT visits | 231 (32.1%) | |  |
| Transportation to DOT visits | 186 (25.8%) | |  |
| Not interested in 3HP - reason unknown | 194 (26.9%) | |  |
| Treatment monitoring reasons: | |  |  |
| *Not interested in coming to clinic for in-person DOT* | | *10* |  |
| *Does not feel comfortable/trusted being observed* | | *1* |  |
| *Need to delay treatment* | | *32* |  |
| *Does not want to wait to start 3HP due to travel* | | *1* |  |
| *In-person DOT will be difficult due to travel* | | *2* |  |
| *Prefers same treatment as family member with LTBI* | | *16* |  |
| *Conflict with drug treatment program restrictions* | | *11* |  |
| *Will be treated through private provider* | | *1* |  |
| *Total:* | | 74 (10.3%) |  |
| Medication-related reasons: | |  |  |
| *Too many pills at once* | | *13* |  |
| *Prefers daily treatment* | | *6* |  |
| *Does not want to change birth control method* | | *5* |  |
| *Prefers other regimen based on past treatment* | | *7* |  |
| *Concerns about side effects* | | *2* |  |
| *Mother’s decision for minor* | | *2* |  |
| *Total:* | | 35 (4.9%) |  |
| **Preferred in-person DOT** | | | **47 (5.9%)** |
| Too many pills for VDOT | | 6 (12.8%) |  |
| Does not want to record videos | | 8 (17.0%) |  |
| Other/Unknown | | 33 (70.2%) |  |
| **Preferred VDOT – refuse to randomize** | | | **8 (1.0%)** |
| **Not interested in being in a study** | | | **25 (3.1%)** |

| **Table S2:** **Percent Effort and Number of Minutes for Personnel to Complete VDOT and DOT Related Tasks by Site** | | | | | | | | | | | | |
| --- | --- | --- | --- | --- | --- | --- | --- | --- | --- | --- | --- | --- |
|  | **East Region** | | **Central Region** | | **North Central** | | **North Coastal** | | **South Region** | | **Student Health** | |
| **Task** | **Effort (%)** | **Min (Range)** | **Effort (%)** | **Min (Range)** | **Effort (%)** | **Min (Range)** | **Effort (%)** | **Min (Range)** | **Effort (%)** | **Min (Range)** | **Effort (%)** | **Min (Range)** |
| Lab Processing | RN: 50  LVN: 50 | 25 (25-45)  25 (25-45) | RN: 100 | 10 (10-15) | LVN: 50 | 10 (10-15) | RN: 50  RN: 50 | 10 (10-20)  10 (10-20) | RN: 50  PHN: 50 | 25 (20-30)  7 (5-10) |  |  |
| Missed appointment | RN: 70  LVN: 30 | 5 (5-15)  5 (5-15) | RN: 100 | 5 (5-10) | LVN: 100 | 8 (2-15) | RN: 90  RN: 10 | 20 (5-20)  20 (5-20) | RN: 100 | 2 (1-4) |  |  |
| Charting patient notes | RN: 50  LVN: 50 | 5 (5-15)  5 (5-15) | RN: 100 | 5 (5-15) | LVN: 100 | 10 (10-15) | RN: 90  RN:10 | 10 (5-10)  10 (5-10) | RN: 50  PHN: 50 | 5 (2-8)  5 (2-5) | RT: 100 | 1 (1-3) |
| In-person DOT visit | RN: 50  LVN: 50 | 15 (15-25)  15 (15-25) | RN: 100 | 5 (5-10) | LVN: 100 | 20 (20-20) | RN: 60  RN: 40 | 25 (15-30)  25 (15-30) | RN: 50  PHN: 50 | 15 (10-30)  15 (10-30) | RT: 100 | 5 (5-10) |
| VDOT set-up | RN: 70  LVN: 30 | 10 (10-15)  10 (10-15) | RN:100 | 15 (15-20) | LVN: 100 | 3 (3-5) | RN: 50  RN: 50 | 15 (10-20)  15 (10-20) | RN: 90  PHN: 10 | 5 (5-10)  8 (8-8) | FC: 100 | 3 (2-5) |
| Watching videos | RN: 70  LVN: 30 | 10 (5-15)  10 (5-15) | RN: 100 | 5 (5-10) | LVN: 100 | 15 (10-20) | RN: 100 | 5 (5-10) | RN: 100 | 10 (6-13) | RT: 100 | 2 (2-3) |
| VDOT closeout | RN: 70  LVN: 30 | 10 (5-10)  10 (5-10) | RN: 100 | 10 (10-15) | LVN: 100 | 3 (3-5) | RN: 100 | 5 (5-10) | RN: 100 | 3 (3-5) | RT: 100 | 2 (2-5) |
| **Abbreviations:** VDOT, video directly observed therapy; DOT, directly observed therapy; LTBI, latent tuberculosis infection; RN, registered nurse; LVN, licensed vocational nurse; PHN, public health nurse; RT, medical resident; FC, field coordinator | | | | | | | | | | | | |

|  | **Table S3. Cost Breakdown for Delivery of 3HP Regimen for LTBI Treatment Using VDOT or DOT** | | | | | | | |
| --- | --- | --- | --- | --- | --- | --- | --- | --- |
|  | | **VDOT** | | |  | **In person DOT** | | |
|  | | **Freq.** | **Unit cost ($)**  **Median (min-max)** | **Total cost ($)^a^**  **Median (min-max)** |  | **Freq.** | **Unit cost ($)**  **Median (min-max)** | **Total cost ($)^a^**  **Median (min-max)** |
| **Personnel costs**  Laboratory  In-person visit  Charting patient notes  Preparing initial LTBI chart  VDOT set-up  VDOT watching videos  VDOT close out  **Subtotal** | | 1  2  12  1  1  12  1 | 10 (10-18)  12 (11-16)  5 (5-10)  12 (8-16)  7 (7-11)  7 (5-11)  4 (3-6) | 10 (10-18)  24 (22-32)  62 (54-125)  12 (8-16)  7 (7-11)  84 (58-133)  4 (3-6)  204 (163-341) |  | 1  12  12  1  -  -  - | 10 (10-18)  12 (11-16)  5 (5-10)  12 (8-16)  -  -  - | 10 (10-18)  144 (133-193)  62 (54-125)  12 (8-16)  -  -  -  229 (206-352) |
| **Equipment costs**  Smartphone^b^  Application^c^  Service^d^  **Subtotal** | | -  1  3 | 13  12  25 | 13  12  75  100 |  | - | - | - |
| **Patient costs** | | 2 | 7 (3-20) | 14 (7-40) |  | 12 | 7 (3-20) | 83 (40-240) |
| **Total cost** | |  |  |  |  |  |  |  |
| with phone and service | | - | - | 318 (270-480) |  | - | - | 312 (246-592) |
| without phone and service | | - | - | 230 (182-393) |  | - | - | - |
| Abbreviations: 3HP, 3-month weekly isoniazid and rifapentine regimen; VDOT, video directly observed therapy; DOT, directly observed therapy; LTBI, latent tuberculosis infection  ^a^ Numbers might not sum to total due to rounding  ^b^ Assuming a $150 smartphone has a lifespan of 3 years and reused 4 times per year  ^c^ Assuming fixed cost for full course of LTBI treatment  ^d^ Assuming 3 months of service | | | | | | | | |

**Figure S1:** Median lower and upper bounds of minutes spent viewing videos based on data captured in the VDOT client management system.
